# Does the prevalence of promotions on foods and beverages vary by product healthiness? A population-based study of household food and drink purchases in New Zealand

**DOI:** 10.1017/S1368980021004936

**Published:** 2022-05

**Authors:** Essa Tawfiq, Kathryn E Bradbury, Cliona Ni Mhurchu

**Affiliations:** 1 National Institute for Health Innovation, Faculty of Medical and Health Sciences, The University of Auckland, Auckland 1010, New Zealand; 2 The George Institute for Global Health, Sydney, Australia; 3 University of New South Wales, Sydney, Australia

**Keywords:** Promotion, Food environment, Household purchasing, Health Star Rating, HSR

## Abstract

**Objective:**

To assess the prevalence of promotions on foods and non-alcoholic drinks purchased by New Zealand households and to determine if they vary according to healthiness of products.

**Design:**

We undertook a cross-sectional analysis of Nielsen New Zealand Homescan® 2018/19 panel data. We conducted multivariate analyses to examine the variability in quantities of healthy *v*. unhealthy food and beverage products purchased on promotion. Promotion was self-reported by the panellist. Healthiness of products was measured by the Health Star Rating (HSR) system. We also carried out a subgroup analysis for beverages according to the threshold of < 5 g *v*. ≥ 5 g sugar per 100 ml content of products.

**Setting:**

The Nielsen New Zealand Homescan® data were linked with two New Zealand Food Composition Databases (Nutritrack and the FOODfiles).

**Participants:**

Food and beverage purchases data by 1800 panel households were used.

**Results:**

Overall, 46 % (1 803 601/3 940 458) of all purchases made were on promotion. Compared with purchases of food and beverage products with HSR < 3·5 (unhealthy), food and beverage products with HSR ≥ 3·5 (healthy) were significantly less likely to be on promotion (OR = 0·78, 95 % CI 0·77, 0·79). The subgroup analysis for beverages shows that products with < 5 g sugar per 100 ml were significantly less likely to be on promotion than those with ≥ 5 g sugar per 100 ml (OR = 0·77, 95 % CI 0·75, 0·79).

**Conclusions:**

Policies to improve healthy food retailing should focus on increasing the promotion of healthier food and drink options in stores and supermarkets.

It is generally accepted that population rises in non-communicable chronic diseases such as type 2 diabetes, obesity, CVD and cancer have been driven mainly by unhealthy food environments^(1,2)^. Unhealthy food environments are determined by the ready availability of affordable and heavily promoted foods and drinks high in salt, saturated fat and sugar^(1,2)^, and increases in food energy supply have been associated with increases in the incidence of non-communicable chronic diseases NCD worldwide^(3)^. Influenced by unhealthy food environments, population overeating of heavily promoted, energy-dense and nutrient-poor food poses a serious global public health concern^(4)^.

Product promotions increase the sales of promoted products^(5,6)^ and lead to stockpiling (buying earlier and/or more than usual) and unplanned purchases of promoted products^(5)^. In this study, we used the general term ‘promotions’ instead of price promotions because households self-reported promotions and it was not possible to determine if products were price promoted or promoted using some other means (e.g. signage, health claims, gifts in the products, characters in the labels or prominent placement in-store). Product promotions influence consumer purchasing behaviours^(7)^ and can increase brand awareness, maintain or improve brand familiarity, generate perceived value and encourage consumers’ positive self-image as bargain shoppers^(7)^. There is some evidence that suggests product promotions increase the volumes of foods or drinks purchased during a single shopping visit and do not lead to reduction in the frequency of purchasing at subsequent shopping visits^(5)^. An industry report in 2018 stated that the proportion of all grocery products sold on promotion was 59 % in New Zealand (NZ), 40 % in Australia, 30 % in the USA, 28 % in Italy, 27 % in the UK, 23 % in Germany, 21 % in Brazil, 20 % in the Netherlands, 20 % in Spain, 18 % in Belgium and 17 % in France^(8)^.

Unhealthy foods and beverages are promoted more often than healthier food and drink options^(9–12)^. A recent study, which used Nielsen New Zealand Homescan® data, October 2016 to October 2017, and examined the prevalence of promotions according to the processing level of food and drink products, found that proportions of unhealthier food options (e.g. ultra-processed and processed foods) promoted were significantly greater than proportions of healthier food options (e.g. unprocessed and ingredient products)^(13)^. In Australia, a recent study that examined the prevalence of beverage price promotions available online at two major Australian supermarket chains (Coles and Woolworths) over 50 weeks, found that within non-alcoholic beverages, the sugar-sweetened beverages (SSB) subcategory had the greatest proportion of price promotions^(14)^.

To our knowledge, in NZ, household self-reported promotions of foods and beverages according to Health Star Rating (HSR) has not been studied so far. In this study, we aimed to examine the prevalence of household self-reported promotion of food and beverage products according to HSR of the products. We used the HSR system, which is an established Australasian nutrient profiling system, to define product healthiness for packaged and unpackaged products. We hypothesised that the prevalence of products purchased on promotion varies according to product healthiness, measured as HSR ≥ 3·5 (healthy) *v*. HSR < 3·5 (unhealthy) for foods and beverages, and measured as < 5 g *v*. ≥ 5 g sugar per 100 ml content of products for beverages.

## Methods

### Study design

This was a cross-sectional analysis of the Nielsen New Zealand Homescan® panel data collected between October 2018 and October 2019. The Nielsen New Zealand Homescan® panel is a sample of 2500 NZ households who are representing NZ households in terms of demographics and geographic locations. We used data from 1800 NZ households as Nielsen New Zealand Homescan® excludes data for households who scan items inconsistently, show sudden changes in scanning behaviour or do not meet the minimum spending criteria. Moreover, data for food purchased at restaurants, takeaways, fast-food outlets and cafés are excluded. The Nielsen Homescan® data is one of the largest commercial food purchasing datasets, and it contains up-to-date data that are used to monitor consumer purchases across twenty-five countries^(15)^. Since Nielsen New Zealand Homescan® data do not include nutrient information, we linked it with two national food composition datasets (Nutritrack and FOODfiles) to extract data on the nutrient profile (energy, total sugar, Na, saturated fat, dietary fibre, protein, Ca and fruit, vegetable, nut, and legume content) of the products purchased.

Data in Nielsen New Zealand Homescan® panel include 1-year household purchases from different food retail stores across NZ. Nielsen New Zealand Homescan® is an open cohort recruiting households continuously, thus accounting for household attrition and limiting demographic changes over time. At the time of recruitment, information on the demographics and geographic locations of the households is collected. The information collected includes the main household shopper’s age and sex, household composition, household size, postcode, and household income. After a household is recruited, the household receives an electronic scanner with a copy of the User Guide on how to use the scanner and collect data on the household purchases for every shopping trip. The panellist household is asked to record food and beverage purchases made at any stores that are taken back into the home. Household data collection takes place continuously as long as a household remains within the panel. A point-earning system is applied to incentivise panel households. Data included in these analyses were collected over a full 12-month period between October 2018 and October 2019. The point-earning system enables conversion of earned points to monetary rewards. After a product is purchased and brought home, the panel member enters the quantity purchased, price of product, whether it was on promotion (yes/no), the store shopped at, and scans the product barcode. The barcode enables the system to derive information on item description, product category, pack size, unit (e.g. g, kg, l and ml), brand and product department. Product departments include beverages, chilled foods, fresh foods, frozen foods, general grocery, and snack foods and confectionary. For purchases of products that do not have barcodes (e.g. fresh produce), the panellist household chooses a corresponding barcode from a supplied booklet. Promotions are self-reported by panellists, which means that promotions are based on the shoppers’ perception as to whether the product purchased was on promotion or not. The panellist households were not provided with a definition of the term ‘promotion’.

For Nutritrack, trained surveyors collect data from four major supermarket chains in the city of Auckland each year. The four supermarkets contain a large range of packaged food and are owned by the two major supermarket retailer companies (Foodstuffs NZ and Woolworths New Zealand) that together hold 89 % of the national grocery market share^(16)^. The trained surveyors use a customised smartphone application which scans the barcode of each packaged food and beverage displaying a Nutrition Information Panel (NIP), available at the time of the survey in the store. Photographs of all surfaces of each surveyed product are taken and uploaded into a web-based database. Nuritrack data include product barcode, product name, food group and category, pack size, recommended serving size, HSR displayed on the product, and NIP information. The NIP includes information on the average amount of energy, total sugar, Na, saturated fat, protein, Ca and dietary fibre per 100 g/ml of each packaged product. This study used Nutritrack 2018 and Nutritrack 2019 data. For unpackaged products, we used FOODfiles. The FOODfiles dataset is the main component of the New Zealand Food Composition Database, which is updated and released online every 2 years. The FOODfiles dataset is the most comprehensive collection of generic food composition data for foods commonly consumed in NZ. We used the most recent FOODfiles database released in 2018^(17)^.

### Exclusion criteria

The food and beverage products eligible for inclusion were those purchased in-store from food stores. This means food and drink products purchased online, at pet stores, at liquor stores and at stores where food forms a small proportion of total sales were ineligible for inclusion in this study. Moreover, products which were purchased infrequently were excluded (less than one unit purchased per month on average across the entire dataset of NZ households). We applied the exclusion criteria at two steps. At step 1, the following products were defined as ineligible and excluded: (i) products purchased at pet stores or stores providing only home delivery or online purchases, (ii) products purchased at stores where food constitutes a small part of total sales (e.g. department stores), (iii) products purchased at stores with no recorded name, (iv) Easter and Christmas products, (v) products not required by regulations to display a NIP (e.g. tea, unflavoured coffee, artificial sweeteners, chewing and bubble gums, gelatine, salt, flour, corn flour, self-raising flour, vinegar, herbs and spices, herb tubes and pastes, cream of tartar, mustard, pepper, baking soda, baking powder, tartaric acid, citric acid, cooking ingredients, ice, curry powder, yeast, bicarbonate of soda, and (vi) special products (baby foods, protein bars, protein powders, and fitness or diet products). Alcoholic beverages and food products purchased at liquor stores were also excluded. At step 2, the infrequently purchased products were excluded. Criteria for infrequently purchased products is described below.

### Data linkage

We used barcode details to link products between Nutritrack and Nielsen New Zealand Homescan®. For those products in Nielsen New Zealand Homescan® which could not be linked in this way, we applied the following four-step approach:

- the products which could not be linked by barcode were listed and ranked based on total units purchased over the 1-year period;
- infrequently purchased products were defined as those bought fewer than 12 units over the 1-year period and were excluded (less than one unit per month on average);
- for fresh produce, FOODfiles was used to extract food composition data, as NIP information for fresh produce is not routinely displayed. For every product, its closest match product was identified by a nutritionist (K.E.B.). The second nutritionist (C.N.M.) resolved issues through discussion when uncertainties regarding appropriate matching arose.
- for the remaining unmatched packaged products, the category-average food composition values were calculated, using the product category nutrient content of Nutritrack products. For example, for a yoghurt product, we assigned the average nutrient composition of all Nutritrack yoghurt products. The nutrient content data used were, energy per 100 g/ml, saturated fat, total sugar, Na, protein, Ca, dietary fibre per 100 g/ml, and the fruit, vegetable, nut, and legume (fvnl) content. Estimated category-level fvnl points data were available in Nutritrack database.

**Fig. 1:**
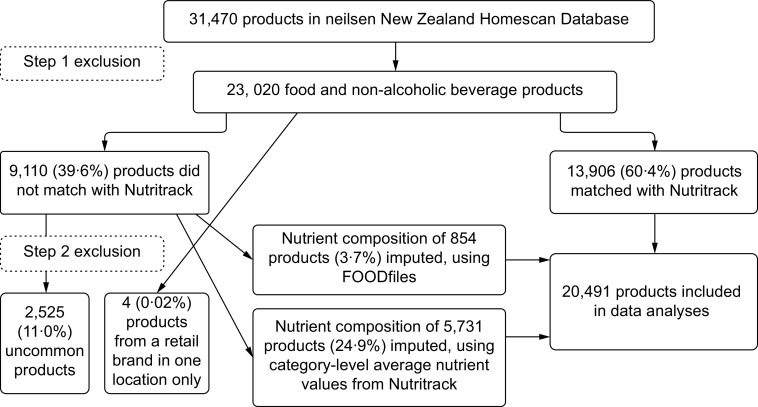
Flow diagram showing number of products included in the study. Note: At step 1, the following products were excluded: (i) products purchased online, (ii) products purchased from stores other than food stores, (iii) products purchased from stores where food forms a small part of total sales, (iv) Easter and Christmas products, (v) products not required by regulations to display a NIP, and (vi) special products. At step 2, the infrequently purchased products were excluded. For details, please see *Exclusion criteria*

Figure 1 illustrates how from an initial total of 31 470 unique products, 20 419 unique products were included for data analyses, after the two-step exclusion criteria were applied. A unique product was defined as a product with a distinctive barcode among all products in the Nielsen New Zealand Homescan® panel data, October 2018–October 2019. This means for the 20 419 unique products included for data analyses, there were 20 419 barcodes available in the database. As Fig. 1 shows, 23 020 products (23 020/31 470 = 73·1 %) were eligible for inclusion after the first exclusion step was applied. Following the second exclusion step, 20 491 (20 491/23 020 = 89·0 %) of all eligible products were matched to food composition data (Nutritrack and FOODfiles) and included in data analyses. In the second exclusion step, 11 % (2525/23,020 = 11 %) were infrequently purchased products, and four unique products were purchased from a retail brand at one location during the 1-year period; therefore, these products were excluded.

### Estimated Health Star Rating

Product healthiness was defined by estimated HSR, which is based on nutrient profiling of products. Nutrient profiling classifies foods according to their food composition^(18)^. Nutrient profiling plays an important role in labelling food and beverage products in the Organisation for Economic Co-operation and Development (OECD) countries^(19–22)^. HSR is a front-of-pack (FOP) nutrition label, and it provides interpretive front-of-pack nutrition information to assist consumers make healthier choices. The HSR system was introduced as a voluntary front-of-pack labelling policy in NZ and Australia in 2014^(23)^. Since it is not required that all packaged products display HSR^(23)^, we estimated HSR for all products (packaged and unpackaged) using the Guide for Industry to the Health Star Rating Calculator^(24)^.

We estimated HSR through four steps. First, we categorised all products into one of six categories: (i) Category 1 (beverages other than dairy beverages), (ii) Category 1D (dairy beverages), (iii) Category 2 (all foods other than those included in Category 1, 1D, 2D, 3 or 3D, (iv) Category 2D (dairy foods other than those included in Category 1D or 3D), (v) Category 3 (edible oil, edible oil spreads, margarine and butter) and (vi) Category 3D (cheese and processed cheese with Ca content > 320 mg/100 g). Second, using per 100 g/ml of energy content (kJ), saturated fat, sugar and Na content of each product, we used the published algorithms^(24)^ and calculated baseline points for all products. Third, using per 100 g/ml of protein content and dietary fibre content of each product, we calculated protein points and dietary fibre points where appropriate. The baseline points were modified by subtracting protein points, dietary fibre and fvnl points from the baseline points. Fourth, using the published algorithms^(24)^, the modified points were transformed into HSR, ranging from 0·5 to 5·0 stars in half-star increments.

### Statistical analyses

We applied generalised linear models (GLM) to examine differences in prevalence of foods purchased on promotion according to product healthiness. The unit of analysis was each unique product purchased, and the outcome variable was a binary response variable as to whether the product purchased was ‘on promotion’ or not. We examined overall estimated mean HSR and percentage of purchases on promotion across the ten categories of product healthiness. The analyses from generalised linear model were adjusted for age of main household shopper (< 34, 35–39, 40–49, 50–65 and > 65 years), sex of household main shopper (male and female), number of family members (1–2 and ≥ 3), equivalised household income level (tertiles of low, medium and high), geographic location (Auckland, Bay of Plenty and Waikato, rest of North Island, Wellington, Canterbury, rest of South Island), product healthiness, product price and store type. Store type was defined as (supermarkets, grocery stores, convenience stores, fruit and vegetable stores, meat and fish stores, and bakeries) using criteria we developed for a recent study (see online supplementary material, Supplemental Table 1). Using the OECD equivalence factors, equivalised household income was generated. This approach for equivalised household income was used in a recent study^(13)^. Household income was categorised based on the midpoint of ten categorical income groups available in the Nielsen New Zealand database. Then the OECD equivalence scales were used to calculate equivalised household income, using the following equivalence factors: 1 for the first adult, 0·5 for each additional adult and 0·3 for each child within the household. The following statistical model was specified for the generalised linear model multivariate analysis:

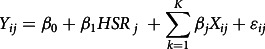





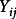
 refers to the binary variable for product *i* (whether promotion equals yes or no), with HSR category of *j*. HSR refers to the binary variable of product healthiness, that is, HSR ≥ 3·5 *v*. HSR < 3·5 stars. 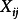 denotes a vector of confounders, and 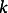 refers to the number of confounders. *β*_0_ stands for the intercept which is odds of the quantity purchases that were made on promotion for products with an HSR < 3·5 stars (reference category), and *β*_1_ refers to odds ratio (OR) of the quantity purchases made on promotion for products with HSR ≥ 3·5 stars. Food and beverage products with a HSR ≥ 3·5 were considered to be ‘healthy’, and products with HSR < 3·5 stars were considered to be ‘unhealthy’. The cut-off of 3·5 stars was based on a technical report showing that this cut-off aligned with the New South Wales healthy food provision policy^(25)^. According to that report products classified as Green by the Traffic Light criteria, on average had a HSR of ≥ 3·5 stars, and products classified as amber or red on average received a HSR < 3·5 stars. We applied the model for all products as well as for non-alcoholic beverages. We categorised non-alcoholic beverages based on sugar content of < 5 g/100 ml *v*. ≥ 5 g/100 ml (reference group). The threshold of 5 g sugar per 100 ml was determined based on the UK Soft Drinks Industry Levy^(26)^. All analyses were performed using STATA version 13.

### Validity of estimated Health Star Rating

Supplemental Table 2 presents the agreement between the displayed HSR and estimated HSR. It shows that out of 2948 products that displayed HSR in Nutritrack dataset, the agreement was 88·2 % and the kappa statistic was 0·74 (*P* < 0·001), showing a substantial level of agreement^(27)^.

## Results

Table 1 shows Nielsen New Zealand Homescan® household demographic and socio-economic characteristics. Out of 1800 households, most of the household main shoppers were in the older age groups of 40–49 years, 50–65 years and > 65 years (86·9 % combined), and most were female (75·8 %). In terms of geographic region, most households were in the North Island (over 75 %) with 29·4 % of these located in the Auckland region. Less than 25 % of households were in South Island with 15·2 % of these in the Canterbury region. The distribution of Nielsen New Zealand Homescan® panel households across the country reflects the population density of the North Island and South Island. Most households consisted of one to two persons (58·2 %), followed by three or more person households (41·8 %). The average monthly household expenditure by store type was highest for supermarkets (median = NZ$446 and mean = NZ$487) and lowest for grocery stores (median = NZ$22 and mean = NZ$32). Product purchases by age group shows that the household main shoppers aged > 65 years had the lowest number of purchases made on promotion (41·9 %), while their purchases were the second highest (1 024 825 units purchased) after those shoppers aged 50–65 years who purchased 1 601 609 units (46·0 % of them on promotion). The age group < 35 years had the second lowest purchases made on promotion (45·1 % of the 220 564 units purchased). The age group 40–49 years had the highest purchases made on promotion (49·0 % of the 794 458 units purchased). Product purchases by income showed that high-income households had the lowest purchases made on promotion (42·5 % of the 1 306 942 units purchased), while low-income households had the highest purchases made on promotion (47·0 % of the 1 299 121 units purchased). The table also shows that 90·0 % of all food and non-alcoholic drink purchases made by the panel were from supermarkets, followed by 7·9 % from fruit and vegetable stores, and the remaining 2·1 % from meat and fish stores, grocery stores, convenience stores, and bakeries combined. In total, there were 3 940 458 product purchases comprising 20 491 unique products. This means for the 20 491 unique products included in this study, on average there were 192 units (3 940 458/20 491) of each unique product purchased by the 1800 households over the 1-year period.

**Table 1:** Demographic and socio-economic status of Nielsen NZ Homescan® panel households, October 2018–October 2019

Household character		Number of households	
Sex of household main shopper	Male	436	24·2 %
	Female	1364	75·8 %
Age of household main shopper	< 35 years	105	5·8 %
	35–39 years	131	7·3 %
	40–49 years	357	19·8 %
	50–65 years	711	39·5 %
	> 65 years	496	27·6 %
Geographic region of households	Auckland	530	29·4 %
	Bay of Plenty and Waikato	292	16·2 %
	Wellington	242	13·5 %
	Rest of North Island	289	16·1 %
	Canterbury	274	15·2 %
	Rest of South Island	173	9·6 %
Equivalised household income	Low-income	643	35·7 %
	Middle-income	553	30·7 %
	High-income	604	33·6 %
Household size (number of persons)	1–2	1047	58·2 %
	≥ 3	753	41·8 %
Monthly household expenditure by store type		Mean NZ$	Median NZ$
	Supermarkets	487·1	445·9
	Meat and fish stores	104·3	78·4
	Fruit and vegetable stores	96·3	71·9
	Convenience stores	57·1	30·0
	Bakeries	34·7	23·5
	Grocery stores	32·1	22·0
Household purchases made on promotion by age group		Units purchased	Percentage on promotion
	< 35 years	220 564	45·1 %
	35–39 years	299 002	47·0 %
	40–49 years	794 458	49·0 %
	50–65 years	1 601 609	46·0 %
	> 65 years	1 024 825	41·9 %
Household purchases made on promotion by income group		Units purchased	Percentage on promotion
	Low-income	1 299 121	47·0 %
	Middle-income	1 334 395	47·8 %
	High-income	1 306 942	42·5 %
Quantities of products purchased at food stores		Units purchased	Percentage of purchases
	Supermarkets	3 545 141	90·0 %
	Fruit and vegetable stores	312 300	7·9 %
	Meat and fish stores	65 198	1·7 %
	Grocery stores	11 468	0·3 %
	Convenience stores	3613	0·1 %
	Bakeries	2738	0·1 %

**Fig. 2:**
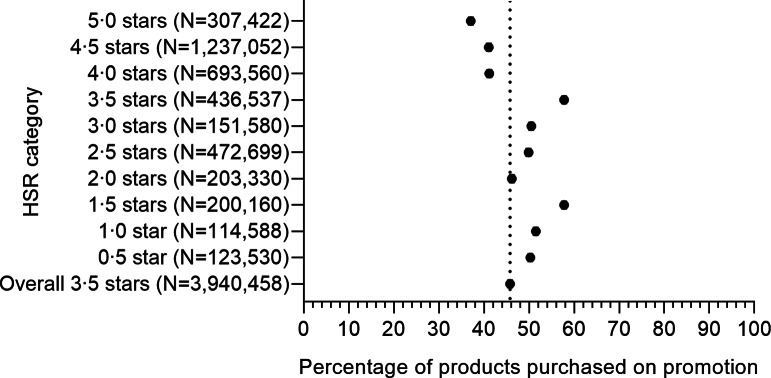
Prevalence of promotions on quantity purchase, by product healthiness. HSR, Health Star Rating

Figure 2 shows the percentage of products purchased on promotion, by ten categories of HSR. Overall, 46 % of all purchases, with a mean HSR of 3·5, were on promotion. Of all 5·0-star products purchased, 37 % were on promotion compared to 41 % of all 4·5-star and 41 % of all 4·0-star products, 58 % of all 3·5-star, 51 % of all 3·0-star, 50 % of all 2·5-star, 46 % of all 2·0-star, 58 % of all 1·5-star, 52 % of all 1·0-star and 50 % of all 0·5-star products.

Table 2 shows the OR of purchasing a product on promotion by healthiness of products. Overall, compared with food and beverage products with an HSR < 3·5, food and beverage products with an HSR ≥ 3·5 were significantly less likely to be on promotion (OR = 0·78, 95 % CI 0·77, 0·79). As for the subgroup analysis, compared with beverages with ≥ 5 g per 100 ml, those with < 5 g per 100 ml were significantly less likely to be on promotion (OR = 0·77, 95 % CI 0·75, 0·79).

**Table 2:** Differences in the prevalence of promotions on quantity purchases, October 2018–October 2019

Food and beverage products *n* 3 940 458	OR	95 % CI	*P*-value
HSR < 3·5 (ref)	1·00		
HSR ≥ 3·5	0·78	0·77, 0·79	<0·001
Beverage products *n* 151 872			
Beverages with ≥ 5 g sugar/100 ml (ref)	1·00		
Beverages with < 5 g sugar/100 ml	0·77	0·75, 0·79	<0·001

Beverage products refer to non-alcoholic beverages.

## Discussion

In this study of annual household purchases by 1800 NZ households, we found that less healthy food was more likely to be purchased on promotion, compared to more healthy food. Overall, compared with purchases of products with HSR < 3·5, purchases of products with HSR ≥ 3·5 were significantly less likely to be on promotion (OR = 0·78). A similar pattern was seen for promotions of beverages where beverages with a sugar content of less than 5 g/100 ml were significantly less likely to be on promotion than those with a higher sugar content (≥ 5 g/100 ml) (OR = 0·77).

Our study is the first to examine the prevalence of household self-reported promotions on purchases of healthy *v*. unhealthy foods and drinks, using 1-year of Nielsen New Zealand Homescan® panel data. Measuring the healthiness of foods and beverages, using the HSR system, and using the sugar content cut-off for beverages is another distinction of our study. In a recent study, for which Nielsen New Zealand Homescan®, October 2016–October 2017, data were used, Zorbas et al.^(13)^ defined healthiness of food and beverage products according to the NOVA system^(28)^. The NOVA system is based on the food processing level (e.g. ultra-processed, processed, unprocessed and ingredients). The authors found that on average 50 % of all annual household grocery items purchased were price promoted. Processed products constituted 59 %, ultra-processed products 55 %, unprocessed products 45 % and ingredient products consisted of 45 % of price promoted purchases. The authors reported that a significantly greater proportion of purchases made by low- and middle-income households were price promoted compared with purchases made by high-income households.

The findings from our study support those from other studies in the Netherlands, UK, Australia and NZ for both food and beverages. In the Netherlands, Ravensbergen et al.^(9)^ studied the prevalence of price promotions for healthy and unhealthy foods, using weekly supermarket flyers over a period of 8 weeks. The authors assessed the product healthiness according to the Dutch ‘Guidelines for Food Choice 2011’ and found greater prevalence of promotions for less healthy products than for healthier products; 70 % of promotions were on unhealthy products. In an Australian study, Riesenberg et al.^(29)^ used online supermarket data on weekly product prices and examined the prevalence of price promotions according to product healthiness, measured by HSR. The authors found that the most price promoted categories during a given week were all discretionary foods (chocolate, 40·3 %; chips, 32·5 %; high-sugar breakfast cereal, 24·0 %; and ice cream, 22·1 %), and the least promoted categories were all core foods (packaged bread, 7·5 %; muesli and oats, 14·7 %; low-sugar breakfast cereals, 15·0 %; and frozen vegetables, 19·2 %). In another Australian study, Zorbas et al.^(14)^ used online supermarket data on weekly product prices from two supermarket chains (Coles and Woolworths) and examined the prevalence of price promotions within different categories of non-alcoholic beverages for 50 weeks. The study found that 26 % and 30 % of all beverages in Coles and Woolworths, respectively, were price promoted in any given week. The authors categorised beverages into four categories: SSB, artificially sweetened beverages (ASB), flavoured milk and 100 % juice, and milk and water. The findings showed that the proportions of price promotions within beverage categories were similar for SSB and ASB (Coles: 30 % of all SSB *v*. 33 % of all ASB; Woolworths: 37 % of all SSB *v*. 38 % of all ASB) and lowest for the ‘milk and water’ category with a weekly average of 14 % for Coles and 15 % for Woolworths.

In a study in NZ, Pollock et al.^(30)^ collected discount information for non-alcoholic beverages from four supermarkets in the Wellington region over a 4-week period in 2008. The authors classified the products into green (drink most), amber (drink in moderation) and red (drink less) categories and examined the percentage of discounts for all three categories of beverages. The authors found that a higher percentage of beverage discounts were for amber (40·9 %) or red (44·1 %) beverages rather than green (14·9 %) across all beverage groups except water (*P* < 0·001). In a UK study, Nakamura et al.^(12)^ used 1 year of household purchasing data from the Kantar Worldpanel survey. The authors defined healthiness of products according to a nutrient profile model endorsed by the UK Food Standards Agency and examined the prevalence of price promotions for healthier compared with less healthy products. The authors found greater prevalence of promotions for less healthy than for healthier foods, after controlling for the reference price, price discount rate and brand-specific effects. There was an increase in sales from 27·3 % to 35·0 % for less healthy products (*P* < 0·01).

The use of nationally representative household food purchasing data collected from a wide range of food stores across NZ over a period of 1 year is a strength of our study. Measuring product healthiness using the HSR system, both for packaged and unpackaged products, including fresh produce, is another strength. This is despite the fact that for some products (e.g. fresh produce), food manufacturers are not required to provide on-pack nutrition information^(23)^, thus making it challenging to use HSR as a measure of product healthiness. In our study, however, using HSR to measure healthiness of packaged and unpackaged products has the advantage that promotion of both healthy and unhealthy products purchased can be examined and compared, and this provides a more complete picture of all food purchased on promotion.

Food policies and interventions can be effective in promoting purchases of healthy foods. Policies and interventions such as increased availability or information as well as monetary incentives for healthy products (e.g. fruits and vegetables) can promote intakes of healthy foods^(31)^. The recent systematic review assessed the effectiveness of food store interventions in promoting consumption of healthy foods^(31)^ and found that in-store health interventions were effective in promoting purchases of healthy foods. According to the review, most of the interventions aimed to increase sales of healthy foods (e.g. whole grains, fruits and vegetables, lower-fat milk, healthier beverages, lower sugar cereals, low-calorie beverages, fish). Most of the studies reported that in-store interventions were effective in one or more of the targeted outcomes^(31)^. Some interventions were single component (e.g. increased availability or accessibility or information intervention), while others were multi-component interventions (e.g. combined information and access/availability or combined monetary incentives and information). The review reported that studies that focused on information provision (in the form of shelf labels, product labels, posters, flyers and distribution of brochures) showed mixed results (e.g. some reported higher sales of promoted food items, while some others reported no difference in sales of promoted products). In contrast, the studies with multi-component interventions reported positive effects in one or more of the planned outcomes, especially increased sales of healthier products^(31)^.

Our study has some limitations. Firstly, although the Nielsen New Zealand Homescan® panel is representative of NZ households in terms of certain demographics (household size and household income) and broad geographical region (upper North Island, lower North Island and South Island), the panel is not recruited to be representative of ethnicity and does not include information on ethnicity of households; thus, we could not adjust our results for ethnicity or report results for ethnicity separately. Secondly, promotion was self-reported by the panellist as a binary response (yes/no). Therefore, in this study, it is not possible to distinguish and examine the prevalence of specific types of promotions (e.g. temporary price reduction, multi-buy offers, larger volumes for the same price as standard volumes, prominent placement in-store, end-of-aisle displays, signage or promotional flyers).

Future research should investigate changes over time in the promotion of healthy and less healthy products, and the impact of any new policy or retailer strategy (e.g. restrictions on promotions of less healthy products) on consumer purchasing behaviour and retailer sales. Another important area with potential policy implications to improve food environments is to examine promotions as well as purchases of healthier compared with less healthy products by neighbourhood deprivation (low/high) and region (urban/rural).

### Policy implications

Our study has the potential to influence food policies and actions to promote healthier food environments. The findings from our study may be used by the government and food industry to increase the availability and promotion of healthy food options across all stores and restrict the availability and promotion of less healthy foods in NZ.

## Conclusion

Based on our findings, nearly half of all products purchased were on promotion. Therefore, compared to healthier food options, it was more likely that greater quantities of less healthy foods were purchased on promotion. To contribute to the reduction and prevention of diet-related chronic diseases, food policies and interventions should focus on increasing the availability and promotion of healthier food options.

## Supplementary material

For supplementary material accompanying this paper visit https://doi.org/10.1017/S1368980021004936. click here to view supplementary material
