# Supplementary material for: Does the prevalence of promotions on foods and beverages vary by product healthiness? A population-based study of household food and drink purchases in New Zealand
Source: Public Health Nutr. 2021 Dec 20;25(5):1246–54. doi: 10.1017/S1368980021004936 (PMC9991816; doi:10.1017/S1368980021004936)
Supplement: Supplementary file 1 [file S1368980021004936sup001.docx]

| Supplementary table 1. Store classification criteria, using Nielsen NZ Homescan® data, Oct 2018 - Oct 2019 | | |
| --- | --- | --- |
| Store type | Criteria | Number of retail brands per category |
| Convenience store  (Service station) | Retails food products,  <10 varieties of fruit and vegetables,  sells fresh milk, and  is not a specialty food store | 9 |
| Grocery store  (Corner store) | Retails food products,  ≥10 - <30 varieties of fruit and vegetables,  sells fresh milk and breads, and  is not a specialty food store | 1 |
| Supermarket | Retails food products,  ≥30 varieties of fruit and vegetables,  sells fresh meats, fresh milk and breads and  is not a specialty food store | 8 |
| Fruit and vegetable store | Retails food products,  ≥ 30 varieties of fruit and vegetables, and  does not sell fresh meats | 4 |
| Meat and fish store | Retails food products, and  ≥ 50% of sales are meat products | 4 |
| Bakery | Retails food products, and  ≥ 50% of sales are bakery products | 2 |

| Supplementary table 2. Agreement of displayed HSR and estimated HSR, Nutritrack 2018 -Nutritrack 2019 | | | | |
| --- | --- | --- | --- | --- |
| Total products | Agree on  healthy products | Agree on  unhealthy products | Agree (overall) | |
|  |  |  |  |  |
| No of products  (%) | No of products  (%) | No of products  (%) | No of products  (%) | Kappa statistic |
| 2,948  (100.0 %) | 1,765  (59.9 %) | 835  (28.3 %) | 2,600  (88.2 %) | 0.74  (P<0.001) |
